# The AI-DRM protocol to enhance the lifetime of wireless sensor network

**DOI:** 10.1016/j.mex.2025.103649

**Published:** 2025-09-25

**Authors:** Santosh Anand, Anantha Narayanan V

**Affiliations:** Department of Computer Science and Engineering, Amrita School of Computing, Coimbatore Amrita Vishwa Vidyapeetham, India

**Keywords:** W.S.N., Sensor, Energy, Lifetime, Prediction model, Propagation model

## Abstract

Energy is a major research challenge in wireless sensor networks since it is placed in an area that is inaccessible to humans. In the current study, nodes send data to their neighboring nodes at any distance using the same energy level. Smaller distances require less energy to transmit to adjacent nodes, creating a strong research gap. High-distance transmissions require more energy. The node must tailor its transmission energy to distance, not fixed energy. The best transmission power for communication is determined via the neural network-based machine learning technique, which is based on the propagation model and network properties, such as the node density, residual energy, and energy harvesting rate. In this work, sensor nodes transmit information to their neighboring nodes via the multiple linear regression model for dynamic radio tuning with the FRIIS propagation model, and the simulation records the node's energy consumption. Compared with the four recent best current methods that increase the W.S.N. lifetime, the proposed protocol is better and uses less power. The proposed AI-DRM protocol has sufficient residual energy to transmit the packet until 1403 rounds, which is higher than those of two recent energy-efficient protocols, the ARORA and the EACHS-B2SPNN protocols.

1. The AI-based dynamic transmission power protocol tunes the sensor nodes using a propagation model.
2. Prediction of lifetime of WSN
3. Effective utilization of all sensor nodes^1^

## Graphical abstract

**Figure:**
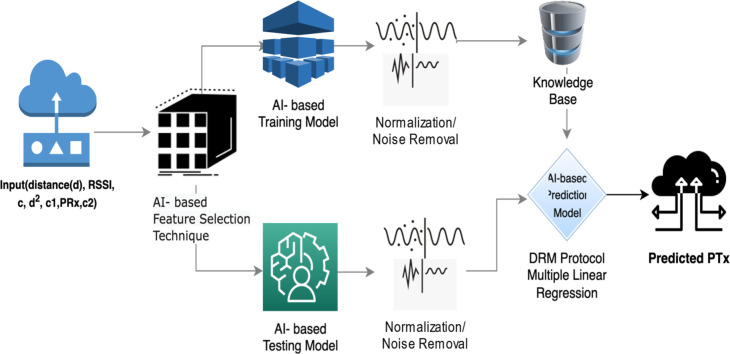

**Specifications table**

**Table:** 

**Subject area**	*wireless sensor network*
**More specific subject area**	*Enhancing the lifetime of WSN*
**Name of your method**	*AI-based Dynamic Radio Management Protocol*
**Name and reference of original method**	*None*
**Resource availability**	*None*

## Background

E. Zhao et al [1] employed the Dynamic Programming approach to distribute the energy efficiency of candidate nodes sporadically across a linear network, aiming to enhance the Network's overall performance. The technique determines the optimal route node and adjusts the transmission range among the considered nodes. Approach is divided into two parts: Part one of the dynamic programming process finds the minimum energy and information, while part two builds the route node. It calculated the energy consumption per second, identified the shortest hops with the least amount of energy consumed, and analysed the energy consumption by sensor nodes near active nodes, among other domain-specific calculations, to obtain these results. One major problem with this work is that it may not be feasible in real-world applications of WSNs, as it requires a significant amount of processing power, which could make it less useful for large-scale, real-time, or resource-limited deployments.

S. Kim and D. Eom [2] offered a reprogramming approach that decreases energy consumption and collisions during message transmission. The authors developed the Dynamic Transmission Power Control (DTPC) protocol to overcome with data loss and data redundancy in WSN. Compared to the MNP protocol, DTPC improves numerous metrics, including energy usage, transmission power, frame inference, packet losses, and retransmission delays. Results from analysing the energy usage of a sensor network environment, as simulated by OMNeT++, were used to evaluate the protocol. The research, dynamic transmission power control introduces additional communication overhead in WSNs and increases delay for packet transmission, which may render its energy-saving benefits less effective in large-scale or time-sensitive deployments.

S. K. A. Imon et al [3] improved the lifetime of WSN by load balancing with minimal temporal complexity using the Randomized Switching to Maximise Lifetime (RaSMaLai) technique. The beneficiary of this work's expansion is the D-RaSMaLai-distributed WSN. The study does not take into consideration the characteristics of the switching probability function in different network settings, which are utilised to accelerate convergence. A major drawback with the RaSMaLai approach is that it can't be used in different scenarios of WSN with different propagation and deployment behaviour.

O. Mokrenko et al [4] reduced the number of active nodes in a WSN to enhance power management in WSNs. The four domains that comprise the subsystems of these sensor nodes are as follows: the power source, the sensing subsystem, the computation subsystem (including memory), and the communication subsystem (utilising radio). The primary goal of the authors is to enhance the battery's power system within the context of its WSN. By activating active nodes and deactivating sleeping ones, Dynamic Power Management technology helps conserve battery energy while mitigating MIPQ problems. One major problem with the predictive dynamic power management scheme in this study is that it employs simple heuristic workload prediction models rather than a more comprehensive system model or one that accurately captures the complex, non-stationary nature of real-world WSN workloads, which makes it harder for the scheme to balance power savings and performance trade-offs reliably.

Santhosh Kumar N and Karthik V [5] applied the New Backbone Scheduling (NBS) method in WSN to improve reliability and longevity. For optimal energy distribution among all sensor nodes, NBS randomly schedules numerous overlapped backbones. This feature allows nodes that aren't involved in the conversation to be put to sleep at random intervals. The backbone network in this work is formed using a Connected Dominating Set (CDS). While designing the route from source to sink, CDS has several disadvantages.

J. Zhang et al [6] implemented a sleeping algorithm that activates the nodes participating in communication and deactivates all other nodes in a WSN. It formulates the Maximum R-hop Connected Partition Lifetime Problem (MRPCL) problem and deploy R-hop CDS Partition Algorithm (RPA) in both backbone scheduling and duty cycling to enhance the lifetime of sensor node and WSN. Yishuo Shi et al [7] described the causes of failures in sensor nodes, with the primary reason being energy (battery or power), as every sensor node works on a small amount of energy supplied by a battery. The author implemented a new protocol to enhance the lifetime of nodes and networks by utilising virtual backbones. Hamza Faheem et al [8] theoretically describe the improvement in lifetime of a WSN using the AODV algorithm instead of CDS. In this, AODV is used to create the path from source to sink, and the VBS sleep scheduling algorithm is used to TURN OFF the other nodes. A major drawback of the implemented a new sleep scheduling algorithm is that it uses the same wake-up intervals for all nodes, regardless of their location or role in the network's connectivity. This means that it doesn't dynamically adjust to changes in forwarding load, burst traffic, or topological importance, which can cause slower performance and increased energy consumption in real-world deployments.

Tian J et al [9] implemented an approach to enhance network connectivity and lifetime by dividing the work into two parts: an algorithm for WSN routing and a novel disjoint set Division (SEDO) algorithm. In a manner analogous to building a backbone network from the ground up, SEDO partitions the Network into smaller networks. As packets travel over the Network, a routing algorithm selects one subnet from the available options. The research disjoint set division algorithm has a big problem: it has only been tested in simulations with static and homogeneous network assumptions. It doesn't take into account changes in the real world, node failures, or mobility, which makes it less reliable and valuable for real-world, heterogeneous WSN deployments.

Q. Yang et al [10] developed the Full Area Coverage Optimisation method to extend the life of WSN in this study. It selects a subset of nodes to communicate with, from the source to the sink, using a probability-based sensing model. Confirming whether a WSN covers a whole continuous area is a challenging task. The primary issue is that probabilistic area coverage strategies often require centralised control and global node information to determine the best activation probabilities, which requires a more substantial amount of time and resources, making them less suitable for large-scale or resource-limited WSN deployments.

C. Sarkar et al [11], in node-scheduling circumstances, Sleeping Beauty surpasses state-of-the-art flooding-based protocols (LWB and FS-LWB) while also being an energy-efficient communication protocol that functions with partial topological information. Energy enhancement is achieved through the following means: 1. An effective neighbor-discovery mechanism that enables the selection of a small, interconnected set of active nodes, and 2. A straightforward yet graceful clock-offset estimation method that allows nodes to sleep for an extended period without requiring explicit resynchronisation. The findings reveal a 5% increase in the number of active nodes in WSN. A significant drawback of this work is that its tightly synchronised slotted communication scheme requires exact time synchronisation and constant neighbour coordination, which increases communication and energy costs.

D. V. Jose and G. Sadashivappa [12] describe WSN as a globally used platform for 'n' number of applications. In this work, the algorithms used are the Artificial Bee Colony Algorithm (ABC), Particle Swarm Optimisation (PSO), and the research technique, which utilises sink contrast with ABC. It resulted in optimal performance, with fewer delays in transmitting packets from the sender to the Base Station (BS) and an average energy consumption. Anuradha Pughat et al [13] compared their work with existing work on WSN to analyse the improvement of WSN's lifetime, where sensor nodes utilise dynamic power management techniques. The MAT lab executes the dynamic power management model using a queue strategy and a first-in, first-out approach. The main problem with the above work is that it was only tested in perfect simulation scenarios, without taking into account real-world issues such as node diversity, environmental interference, or scalability in large-scale WSNs.

Bilquis Amaliah et al [14] evaluated the network's lifetime by estimating optimal values using various mathematical formulas. In this improved version of Dijkstra's algorithm, it was implemented on Java Island to save energy and reduce the distance between locations. It achieves 92.88% accuracy compared to Google Maps. Limitation: The algorithm requires more memory, which complicates the operation of the WSN. In this work, deleting nearest-neighbour nodes can degrade the accuracy of WSN shortest paths in about 7% of cases, which also leads to unreliable communication.

P. P. Priyeshand and S. K. Bharti's [15] goal is to decrease energy consumption during node data transmission without compromising communication link quality. It is necessary to conserve energy by minimising uncontrolled power transfer to lower the consumption rate, as the batteries used by the sensor nodes are smaller and have a shorter lifespan. The techniques for managing transmission power include the Received Signal Strength Indicator (RSSI) and Proportional Integral Derivative (PID) control deployed in WSN with the closed-loop feedback method. A significant issue with this work is that it requires careful tuning of control gains, which can lead to instability due to variations in channel conditions or nonlinearities that occur, making it less reliable in real-world deployments.

Mohamed Elshrkawey et al [16] performed the study by grouping the sensor nodes into clusters, with one key node designated as the Cluster Head. The CH communicates with the base station by collecting data sensed or made through comparisons with the sensor nodes (SNs). In the research, the authors compared the energy consumption and performance of the transmitting nodes to that of the LEACH protocol and effectively addressed the three research challenges of LEACH. The authors implemented using MATLAB 2015a with an Elliptical Gaussian distribution, which extends the LEACH protocol's network life cycle by 60%. This work doesn’t consider other parameters of the network, such as node density, link quality, and other overhead in WSN.

D Lubin et al [17] give a comprehensive analysis of WSN applications that can be built with or without infrastructure. To improve the system's efficiency, sensor networks utilise the dynamic programming approach to address challenges in routing algorithms. When compared to the current WSN routing method, the results show a significant improvement in energy efficiency. The primary drawback of this survey is that it relies solely on simulation-based evaluations and doesn't include any studies on the real-world applications of evolutionary algorithms for energy efficiency in WSNs.

Daanoune et al [18] discussed the role of the WSN in the current generation, focusing on data transfer, communication protocols, and the constraints of a battery's power source. With an emphasis on Enhanced Energy, this researcher developed an improved LEACH Routing Protocol that aids in reducing power usage during short-distance data transmissions from nodes to sinks. These days, sensor nodes are used for environmental monitoring, and these sensor nodes have limited energy. Hence, it is not possible to recharge or replace the sensor nodes. The research work is implemented using MATLAB2016b and shows better energy enhancement compared to the LEACH with parameters of number of sensor nodes and sink. One of the most significant challenges of this protocol is that it typically makes things more complicated and consumes more resources because it updates routes so frequently. This could make networks less scalable and less energy-efficient in big or very dynamic WSN situations.

Safa S. Saleh et al [19] aim to enhance the performance and lifespan of WSNs by employing low-power consumption for CH selection, thereby improving the LEACH protocol. A significant issue with the LEACH protocol is that it doesn't choose the CH at random. The suggested protocol, IE2-LEACH, employs a reliable method to identify cluster markers. This method prioritises the most critical aspects: reducing the number of sensor nodes, eliminating duplicate data, and ensuring that energy consumption is evenly distributed among the headers. This makes the selection process better than random selection. This protocol does not consider the real factors such as changing link reliability, deployment diversity, and synchronisation overhead.

N. C. Resmi and S. Chouhan [20] identified erroneous bits and also reduced transmission energy. The purpose of this is to prevent the energy-wasting transmission of erroneous bits. It presents a more effective approach to implementing energy-efficient interdependent source–channel coding in WSNs. It combines compression and error-control coding to reduce unnecessary transmissions while enhancing bit-error performance. Farman Ullah et al [21] improved the energy efficiency of the routing protocol and utilised stable resources in the Wireless Body Area Network (WBAN) with the deployment of ERRS, which stands for Energy-Efficient and Reliable Routing Scheme. Using the forwarder node rotation technique works deployed for the sensor node selection communication. It can't be used in the real world, as it only works with perfect channel models and sensor nodes that are all the same. It doesn't consider factors such as fading, interference, or hardware differences.

D. Vimala & K. Manikandan [22] compared a large number of sensor nodes to the base station in terms of data collection, processing, and transmission to neighbouring nodes. It introduces a new paradigm for optimising the energy efficiency of WSNs across layers, with a focus on Grid-Based Energy-Efficiency Cross-Layer Optimization (GEECLO), Grid partitioning, clustering, and routing are the primary components of this approach. In this, Dolphin Swarm Optimisation Algorithm (DOSA) handles the routing process of selecting the optimal route for communication. The GEECLO routing approach is employed to prolong the life of the WSN and improve its energy efficiency. The experimental results demonstrate that by selecting the appropriate cluster heads and utilising GEECLO for efficient communication routing, a substantial amount of energy is saved. This method can cause cells to use energy unevenly and is impractical in WSN scenarios where resources are limited or change rapidly.

A. Esse et al [23] focused on enhancing the energy efficiency of the routing scheme and also addressed the stability issue caused by resource limitations in the wireless body area network. It works by selecting the nodes using node selection and node rotation techniques. A significant issue with the dynamic power splitting SWIPT receiver is that it assumes an idealised linear energy-harvesting model and perfect control over splitter ratios, which could lead to overestimating energy gains in real-world WSN deployments.

Ren Xiaojun et al [24] achieved energy conservation by partitioning the energy and employing dynamic power transfer. Here, energy conservation is accomplished by utilising radio frequency and adjusting the power splitting between sensor nodes. The results of this work are compared with those of DPA-SWIPT and other power signals and ranges that achieve excellent throughput. In this work, real time parameters are not considered.

Alabady et al [25] designed an enhanced energy optimisation routing protocol for WSNs to extend the lifetime of sensor nodes by utilising a CH selection methodology. The research protocol in this work combines three different clustering routing protocols, utilising only their advantages, with the Enhanced Energy Optimization Routing Protocol. This protocol primarily operates in the domains of intra- and inter-cluster data forwarding, which also uses the scheme for calculating the optimal distance between cluster heads. The results also show that the maximum energy saved is achieved by balancing energy utilisation and extending the Network's lifetime. A drawback of this work is that it only considers residual energy and distance, which means it may overlook other essential aspects, such as node density, traffic load, and link quality, that could impact the network's overall energy efficiency and performance.

Kalpna Guleria et al [26] enhanced the LEACH technique to conserve a lesser amount of energy, depending on residual energy and distance. The research protocol is to Enhance energy conservation based on residual energy and distance protocol. The primary objectives are to conserve energy, minimise the time required for exchanging data packets between clusters, balance the load across nodes, and enhance the Network's longevity. The strategy used to solve this problem involves selecting the accurate cluster head based on residual energy and calculating the distance when choosing the path using the multi-hop technique. The results of simulating research work yield improved outcomes compared to all other LEACH protocols. It might add more computational burden and complexity to heterogeneous WSNs, which could hurt real-time performance and scalability.

Battina Srinuvasu Kumar [27] implemented a clustering methodology to reduce energy utilisation in sensor nodes by tracking events. Here, static and wireless nodes are used to build the Network. Initially, these static nodes broadcast information, after which wireless nodes select a cluster head from among them. Based on its position and energy level, the wireless node selects the cluster head and then sends the data to it. This work also introduces the concept of locating stationary relay nodes to facilitate the computation of the optimal solution for sensor nodes. The enhanced energy-efficient clustering algorithm yields results with minimal energy consumption compared to existing algorithms, thereby extending the Network's lifespan. It may take a considerable amount of time to compute and fine-tune the parameters, which could render it less valid in WSN situations where time or resources are limited.

Soleymani et al [28] implemented a methodology related to a multi-hop routing protocol, which differentiates routing between the cluster head and the base station. Both the selection of cluster heads and the distance between nodes lead to increased energy usage. This work addresses these issues by modifying the genetic algorithm, which transmits data directly to the sensor nodes. The alteration of selecting the cluster head is also done to decrease energy consumption. This work is simulated in MATLAB, displaying results with effective and efficient outputs compared to other existing protocols. The accuracy of the research work depends significantly on the quality and consistency of the previous data. This data may not always be available or reliable in dynamic WSN systems.

Lama A. et al [29] introduced a hybrid data prediction approach that leverages three techniques: Kalman Filtering (KF), Decision Tree (DT), and the autoregressive integrated moving average (ARIMA) model. KF is a preprocessing step used to filter out noise and redundancy from raw sensor node data, allowing only significant data points to be utilised for estimating sensor node readings and optimising prediction accuracy in clustering. Cluster heads are dynamically selected, and sensor nodes are organised based on data analysis that considers spatial and temporal correlations in the data, including energy, location, and data similarity. The ARIMA model is employed to predict future sensor node values based on past data trends, facilitating time-series prediction. If the expected value is within an acceptable threshold (error bound ε), the data is not transmitted. If the deviation exceeds ε, the updated data is sent to the base station. Only necessary transmissions occur, reducing network traffic. The model provides cluster-based data prediction, minimising transmission overhead. It enhances prediction accuracy by self-tuning Kalman filtering while minimising computational costs. It reduces the number of transmitted packets by forecasting data trends using energy-efficient sampling. It requires a significant amount of computing power. It is sensitive to previous data selections, which can make it less scalable and less accurate when working with large or highly variable datasets.

Nadia Khiadani and Faramarz Hendessi [30] developed an energy-efficient target-tracking protocol that utilises Kalman filtering for precise position estimation and prediction, thereby extending the network lifetime by minimising unnecessary communication and optimising sensor node activation and scheduling. Sensor nodes are randomly distributed throughout the deployment area, with each sensor node having location and energy level data of other nodes to create an awareness map. When the target is sensed in the deployment area, the node with the highest energy level is selected as the leader, assisted by two nodes with the second and third-highest energy levels, respectively, which estimate the target's position. The leader node aggregates data from assistant nodes. It applies KF to assess the current position of the target and predict its future positions, which are updated periodically based on new data. When the target leaves the leader's sensing range, the system elects a new leader node. In the system, only three nodes are active at any given time, thereby minimising communication overhead and reducing unnecessary transmissions.

T. De Sales Bezerra et al [31] analysed various radio wave propagation models and identified the most suitable model for water reservoirs. The signal quality of the models is assessed using the RSSI, Mean Square Error (MSE), and Mean Absolute Percentage Error (MAPE) values. The study was conducted for a water reservoir in Campina Grande, Brazil. While the receiver module travelled along the reservoir, the transmitter module remained stationary. One hundred samples of RSSI values were collected at 10 different locations. In the water reservoir setup, the Free Space Model provided the best accuracy, with the lowest MSE and MAPE values among all the analysed and experimented models, thanks to the open and reflective nature of the environment. The primary limitation of this work is that it utilises propagation models that may not fully capture the complex and dynamic conditions in water reservoirs, which may lead to less accurate predictions when applied in the real world.

AL-Shukrawi et al [32] explored the Q-learning algorithm aimed to develop an AI-based energy optimisation technique for adaptive routing and effective management of energy resources. The algorithm enables sensor nodes to autonomously learn optimal energy management strategies and adapt routing based on real-time network conditions, minimising energy wastage. Sensor nodes are randomly deployed in the sensing area, where each sensor node measures and exchanges network information, including energy and connectivity, to establish an initial energy map. The state of each node is represented based on its energy level, traffic load, and connectivity, which is dynamically updated in response to real-time network changes. Nodes select actions (adjusting transmission power, switching routing paths, or entering sleep mode) using the Q-learning algorithm and follow the reward function. The system updates the Q-values for every state-action pair based on real-time network feedback, awarding positive rewards for energy-efficient choices and negative rewards for inefficient routing or high energy consumption. Nodes dynamically select the most energy-efficient paths for data transmission based on real-time Q-values. When not actively sending data, the system can enter low-power sleep mode and independently adjust its transmission power based on network conditions. Yash Goswami et al [33] forecast energy consumption data in WSNs using deep learning techniques. In this work, sensor nodes forecast the required energy and residual energy, and an artificial neural network uses the energy utilisation multilayer pattern to predict the next round of energy needed. Aruna Reddy H et al [34] explored the use of deep learning and machine learning techniques, specifically Particle Filter (PF), Long Short-Term Memory (LSTM) networks, and Random Forest (RF), to predict energy efficiency in WSNs and optimise power consumption. The study proposes a hybrid approach combining the three techniques to optimise predictions. PF is used for data filtering, eliminating redundant information, and optimising transmission paths. RF technique is used to organise sensor nodes into logical clusters. At the same time, LSTM is used to analyse and predict energy usage patterns based on past values. Brijesh L. Kundaliya et al [35] provide a detailed survey of the application of machine learning in WSNs. The survey aims to research ML-based solutions addressing key challenges in WSNs like energy consumption, network routing, node participation, and fault tolerance. ML is categorised into supervised, unsupervised, semi-supervised, and reinforcement learning, and algorithms such as K-Nearest Neighbour (KNN), Decision Trees, Artificial Neural Networks (ANN), Support Vector Machines (SVM), Regression, and Q-learning are thoroughly explored, enabling data-driven decision-making in WSNs, providing significant improvements in network adaptability, and reducing operational overheads. Wang Y. et al [36] explored clustering techniques that utilise machine learning algorithms, such as K-means, Fuzzy C-means, and hierarchical clustering. These techniques support energy-aware task allocation, node grouping and routing, and reinforcement learning that adapts node behaviour based on environmental conditions. The paper is a comprehensive review, but it does not provide experimental simulations or case studies to analyse the concepts introduced in depth. Machine learning also presents new challenges in WSNs, including data noise, training complexity, and hardware constraints. The problem lies in the training data of AI models, which requires extensive computing power, a resource that may not be feasible for wireless sensor network nodes with limited capabilities.

Namita Shinde and Dr Vinod H. Patil [37] deployed the Coot Optimization Algorithm (COA) in WSN to address the research challenges of energy, delay, and security. The advanced version of the fitness function for CH selection and path selection is based on several factors, such as distance, trust, security, delay, residual energy, and load management. The result shows a better lifetime of up to 3571 rounds with 0.8 security and 0.6 trust. The coot algorithm may take a long time to converge or become stuck in local optima, which could lead to suboptimal energy management in large-scale or highly dynamic WSN systems.

L. Sahoo et al [38] select CH based on the integration of intelligent systems and multi-criteria decision-making (MCDM) in uncertain environmental parameters to enhance the lifetime of WSN. A smart system utilises the silhouette (SI) score in conjunction with the Density-Based Spatial Clustering of Applications with Noise (DBSCAN) algorithm to dynamically select cluster heads. Additionally, MCDM (Multi-Criteria Decision Making) is employed to choose energy-efficient cluster heads for communication. The research simulation results indicate that the WSN lifetime improved by 43%, 38%, and 15% compared to HEED, REACT-IN, and LEACH-FC, respectively, using triangular fuzzy numbers (TFN). If uncertain parameters are not handled correctly, the intelligent clustering approach may not function optimally, and the network may not last as long.

M. R. Senkumar et al [39] deployed a custom-made, energy-efficient routing protocol for selecting the CH in WSN. It follows three steps: 1. K-means for cluster formation and CH selection, 2. Adaptive neuro-fuzzy system for nomination of super CH, and 3. Black Widow optimisation for best path selection. The technique enhances network lifetime and energy efficiency; however, its limitations overlook node mobility, varying energy levels, and real-time communication constraints, which limit its applicability in dynamic or mission-critical WSN installations Xiaogang Y [40] deployed Secure Low-Energy Routing Protocol (SLERP) to dynamically adjust trust values and achieve load balancing in the network, thereby ensuring secure communication while reducing the presence of malicious nodes in the network using a Chebyshev neural network. Dynamic trust evaluation and energy balance across nodes improve security in WSN. Still, the limitation of the protocol adds computational and communication overhead that can impair efficiency and scalability in large-scale or severely resource-constrained WSNs. Lei et al [41] implemented a novel hybrid energy-aware routing protocol in WSN to enhance energy and throughput. Fuzzy clustering and PSO enhance cluster stability and energy efficiency; however, this work's limitations include delayed convergence and higher computational complexity, rendering them unsuitable for large-scale or real-time IoT installations with strict latency requirements. Othman S [42], a unique Decentralised lightweight group key management (DLGKM) framework is integrated into Reliable & Secure multitasking routing protocol (REMI) to provide energy-efficient, better packet delivery by 99.21 % compared to the existing work quality-based multicast routing (QMR), Secure routing protocol for low power and lossy network (SRPL) and Mobile AODV (MAODV). Group key management enhances security, but its limitations become apparent when key distribution and updates require communication and processing, which can strain low-power IoT devices and compromise scalability in large or dynamic networks. Ahmed S [43] implemented a Bio-Inspired BOA-PSO algorithm, Butterfly optimisation algorithm (BOA) performs the cluster formation and CH selection, and Particle Swarm Optimisation (PSO) selects the optimal path for communication from source to CH and CH to sink. In fast-changing disaster scenarios, the bio-inspired technique enhances energy efficiency and adaptability; however, its limitations arise during high node mobility, irregular topological changes, and real-time response requirements.

Literature survey: In this [53] research work, the authors implemented an innovative framework that integrates Deep Learning-Enhanced Hybrid Trust (DLEHT) and Machine Learning-Enhanced Fuzzy-Based Routing Protocol (ML-EFBRP) to enhance lifetime, reliability, and security in WSNs. Probabilistic models are deployed to analyze past behavior to identify rogue nodes, enhance throughput, and reduce latency and packet loss. In this research [52], the authors investigate the integration of a Time Splitter Method with RF-based energy harvesting to enhance power management in wireless sensor networks. The study demonstrates that utilizing a simulated helical antenna, combined with mathematical modeling and free-space propagation, yields improved energy efficiency and reduced noise and path loss.

Research Gap: Based on the discussion in Section 2 [[1], [2], [3], [4], [5], [6], [7], [8], [9], [10], [11], [12], [13], [14], [15], [16], [17], [18], [19], [20], [21], [22], [23], [24], [25], [26], [27], [28], [29], [30], [31], [32], [33], [34], [35], [36], [37], [38], [39], [40], [41], [42], [43]], authors have designed and developed routing protocols, sleep techniques, path selection, path optimisation, cluster head selection, and artificial intelligence to extend the lifespan of WSNs. These works face several challenges, including varying energy levels, issues during node mobility, being unsuitable for dynamic or mission-oriented tasks, high latency, strict latency requirements, and security vulnerabilities. The authors must construct real-time hardware and verify the simulation with hardware data. Since nodes are randomly deployed, authors should focus on the total energy of all nodes rather than specific paths. The source node in a WSN must communicate data to its destination, sink, or base station (BS) via trusted neighbour nodes after deployment. Sensor nodes require transmission energy to send data to neighbouring nodes, their neighbour's neighbours, or the base station. A significant drawback of existing works is that they employ a fixed transmission power independent of data size and distance, which results in increased energy wastage at nodes and a reduced lifetime for WSNs.

## Method details

**Fig. 1:**
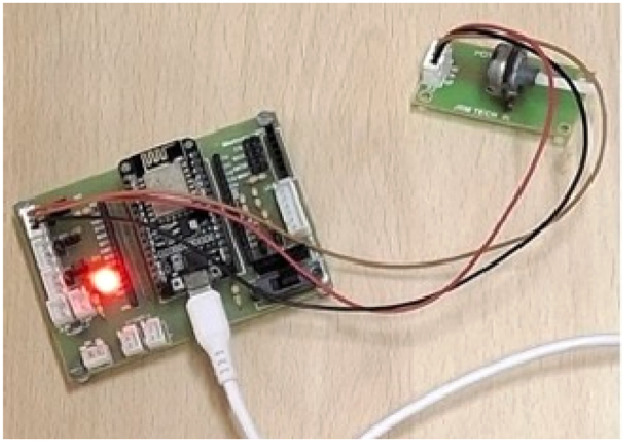
Mobile sensor node.

**Fig. 2:**
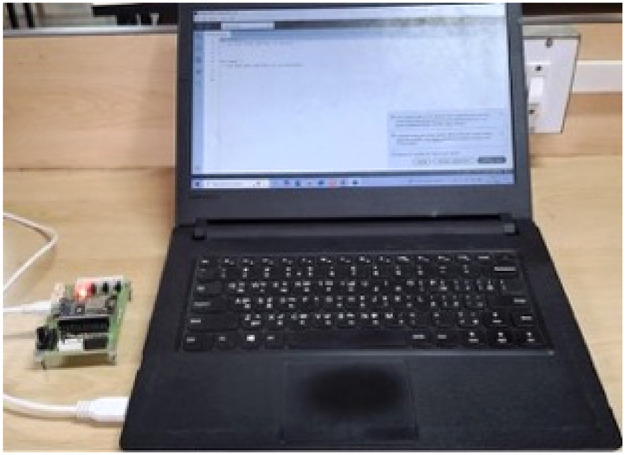
Sink node.

WSNs can be used for important tracking and monitoring tasks, such as border, sea, and forest monitoring. Because WSNs are deployed in areas that are inaccessible to humans and their sensors run on batteries, their lifespan presents significant research challenges. Therefore, recharging and replacement of the battery are not possible. This WSN requires a protocol that can efficiently use the energy of all sensor nodes without wasting it because the nodes are deployed at random. In the current WSN scenario, all the sensor nodes consume identical amounts of energy without concentrating on the distance from the next node or the packet size. In this work, two sensor nodes are created using microcontrollers and other hardware to collect real-time data for the prediction of transmission power, and the prediction technique is applied while simulating the WSN. The RSSI values are measured while moving both nodes at different distances via the Arduino IDE’s COM3 port. The mobile sensor nodes are shown in Fig. 1 below, and the sink node is shown in Fig. 2. When the distance between nodes increases, the signal strength (RSSI) decreases. In the proposed protocol, the collection of real-time RSSI data plays a major role in the prediction and simulation of the WSN to increase its lifetime.

**Fig. 3:**
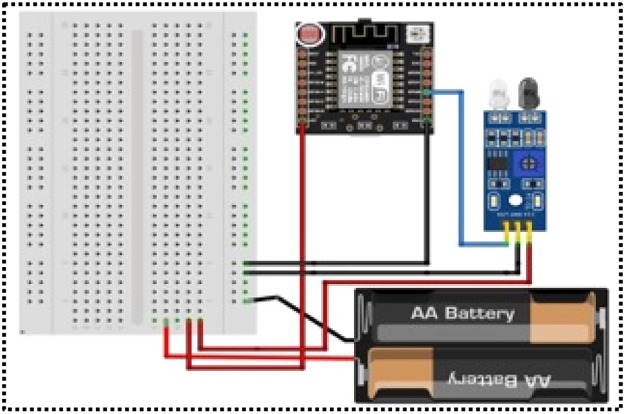
Schematic diagram of Sensor Node Figure.

**Fig. 4:**
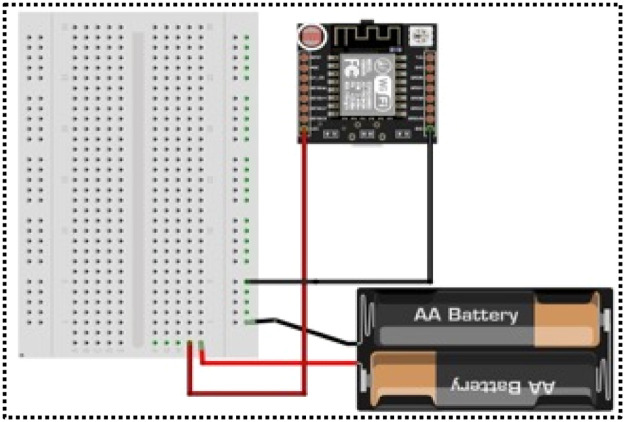
Schematic diagram of Sink Node.

**Fig. 5:**
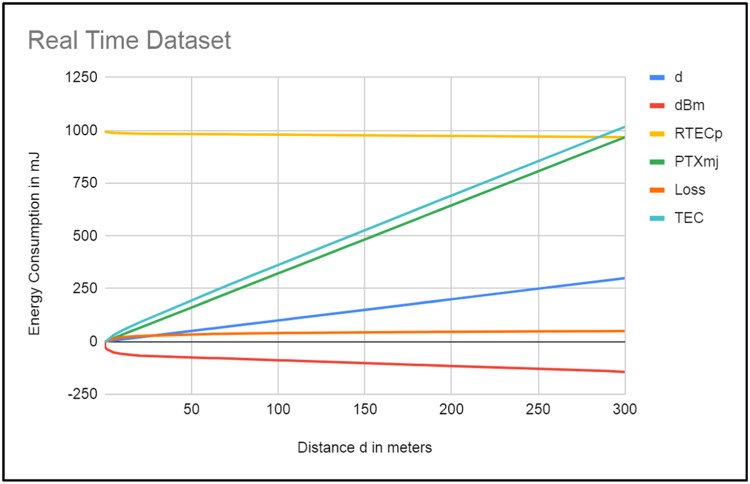
Energy calculations and observations within 300 m.

**Customised Prototype:** The prototype for the sink node and sensor node, shown in Fig. 1, Fig. 2, is a customised prototype designed using the Fritzing non-profit organisation application (Fig. 3, Fig. 4) and developed in the laboratory to meet the research objectives. These prototypes make a major contribution to fetching real-time datasets and tuning sensor nodes to consume only the required energy for transmission, based on the data size and distance.The sensor node prototype is powered by a battery and can operate over different distances ‘d’. The sink node prototype has an unlimited power supply and works as a base station. The sink node can observe and collect the signal strength between nodes at different distances ‘d’. The transmitter signal is measured at different distances and with various obstacles between source node to its neighbour nodes. Initially, the RSSI strength of the transmitter is measured with distance ‘d’ without any obstacles. It shows variation of signal strength when distance changes. When there is no obstacle in the communication path between transmitter and receiver, a transmitter can communicate with a maximum distance of 300 meters, as shown in Fig. 5.

The embedded system, featuring a Node MCU, is utilised in the WSNs to obtain the RSSI value related to distance, which primarily used in Dynamic Radio Management. Schematic designs of the prototype sensor node and sink node are illustrated in Fig. 3, Fig. 4, respectively, and programmed with mathematical models for the Framework.Schematic design of the Hardware Prototype (microcontroller attached to an ESP32 module) for the sensor and sink node, helped to fetch the RSSI values. The communication pins (TX and RX) and General-purpose input/output (GPIO) for sensor inputs are marked. It runs on batteries and features live RSSI readouts, utilising an IDE and a Secure Digital card device that logs signal strength across various distances. The design flow goes logically from power input to data capture and storage. The parts are spaced out so that they may be easily aligned on a breadboard or miniaturised on a PCB in the future. To make the setup easier and reading easier, key pins and connections are colour-coded.

### Data collection and assessment via the propagation model

#### Parameters of the proposed model

SN: Sensor Node Type; d: Distance between nodes; RTECp: Real-Time Energy Consumption per packet; RTEXap: Real-Time Energy Consumption for all packets; PTXmj: Actual PTx m joule per packet; PTXAPmj: Actual PTx m joule for all packets; FPTx: Fixed power for receiving 30 millijoules of 5 meters. TEC: Total energy consumption. The AI-based dynamic radio management protocol (DRMP) receives the data gathered from the hardware, such as d, RSSI, and RTEC, as shown in Fig. 3 below (the graph is generated from the original dataset to reduce the number of pages). The RSSI value of the mobile node has been observed at the fixed node. while the other node moves with distance d. These RSSI values are then converted into milliwatts and from milliwatts to millijoules. The RTECp values in Fig. 3 decrease as the distance between nodes increases. However, in the actual case, it should be in reverse order when the distance between nodes increases. Nodes have to use more transmission energy for data transmission.

**Role of the FRIIS Propagation Model:** In the proposed protocol, nodes are tuned according to the propagation model. The energy consumption of the node is reduced when the distance decreases, which can result in an increase in the lifetime of the W.S.N., as shown in the TEC column. The outcome demonstrates that, prior to network deployment, dynamic radio tuning can be applied to the sensor via embedded or alternative programming on the chip. Then, the lifetime of W.S.N. can be increased.

#### Architecture of AI-based DRM

Below, Fig. 4 shows the input of the proposed DRM model as described in Section 3.1 and implemented in Section 6.1. Fig. 5 shows the training, implemented in Section 6.1, and testing, implemented in Section 6.2, of the DRM protocol, which is implemented in Section 6.

#### Algorithm for the dynamic radio energy consumption of a WSN

In the proposed DRM algorithm, all the sensor nodes are tuned with the FRIIS propagation model to consume the PTX, as shown in Fig. 7, which is not static and depends on the distance between the next sensor or sink. Below algorithm, Step 1: Calculate the distance of all sensor nodes to their neighbour nodes. Apply the FRIIS propagation model to calculate the real-time required transmission power with its neighbour nodes. Step 3: Check threshold values and residual energy level, apply required PTx. Step 4: Energy consumption when no event occurs, Energy consumption while the sensor senses the upcoming event, Energy consumption while transmitting data to the next node, Energy consumption per bit of packet size. Step 5: Deploy a regression model for the energy consumption of the sensor node. Step 6: Calculate the total energy consumption of sensor nodes in WSN.

Selecting a prediction model to predict the PTX with the propagation model is challenging in the current era of heterogeneous sensors in WSNs. Therefore, three prediction models—1. Random forest regression, 2. Decision Tree Regression, and 3. Multiple linear regressions are applied to the dataset in this proposed DRM model to predict the necessary total energy consumption (TEC). In the WSN dynamic energy consumption prediction dataset, the multiple linear regression model is more efficient and consumes less energy than the other two models. It also has a lower error rate. Therefore, in the DRM model, a multiple linear regression model is used, which is shown in Fig. 5.

### The AI-based DRM model

#### Training model for the AI-based DRM model

Step 1. It is necessary to forecast transmission power in relation to distance in this proposed work. Therefore, the inputs are given as all the parameters obtained from the W.S.N., such as the distance, distance square, RSSI, actual PRx, constants (c1 & c2), and PTx. When independent data (feature values) are used, the algorithm will choose dependent data (predicted values). It provides the feature values as distance in meters (d), RSSI in dBm (s), distance square (d2), actual PRx in joules per second (r), actual PRx with constant in joules per second (rc), and the required predicted values as PTx in joules per second (t).

Step 2. These values are then sent to the classifier-based feature selection technique to obtain the best features from the available features. The feature selection techniques select the five best features: distance in meters, RSSI in dBm, actual PRx in joules per second, and constant 1. Among these features, four are more dependent.

Step 3. After the best feature selection, noise removal techniques, such as NULL values and high-variant data (outliers), are applied to remove unwanted data from the feature matrix. Because some sensors may not respond correctly, some may be given high values due to the environment. Therefore, these types of sensors produce null or outlier results.

Step 4. The correlations between features and predicted values were calculated via the Pearson coefficient of correlation statistical method via Equations (1) to (5).

(1) $${ß}_{1}=\;\frac{\sum_{i=0}^{m}\left(d_{i}-\bar{d}\;\right)\left(t_{i}-\bar{t}\right)\;}{\sum_{i=0}^{n}{\left(d_{i}-\bar{d}\;\right)}^{2}}$$

(2) $${ß}_{2}=\;\frac{\sum_{i=0}^{m}\left(s_{i}-\bar{s}\;\right)\left(t_{i}-\bar{t}\right)\;}{\sum_{i=0}^{n}{\left(s_{i}-\bar{s}\;\right)}^{2}}$$

(3) $${ß}_{3}=\;\frac{\sum_{i=0}^{m}\left(ds_{i}-\bar{ds}\;\right)\left(t_{i}-\bar{t}\right)\;}{\sum_{i=0}^{n}{\left(ds_{i}-\bar{ds}\;\right)}^{2}}$$

(4) $${ß}_{4}=\;\frac{\sum_{i=0}^{m}\left(rc_{i}-\bar{rc}\;\right)\left(t_{i}-\bar{t}\right)\;}{\sum_{i=0}^{n}{\left(rc_{i}-\bar{rc}\;\right)}^{2}}$$

(5) $${ß}_{5}=\;\frac{\sum_{i=0}^{m}\left(r_{i}-\bar{r}\;\right)\left(t_{i}-\bar{t}\right)\;}{\sum_{i=0}^{n}{\left(r_{i}-\bar{r}\;\right)}^{2}}$$

where ß1, ß2, ß3, ß4, and ß5 are Pearson coefficients of correlation, $x_{i}$ are independent values, $\underset{‾}{x}$ = x means, $y_{i}$=dependent values, and $\underset{‾}{y}$ = y means.

Step 5. The slope between -1 and +1 is provided by correlation values.

Step 6. The y-intercept is calculated on the basis of the slope via the equation 6 to obtain the starting point of the slope.

(6) $$\underset{‾}{ß0\;=\;y}\;-\;ß1\;\underset{‾}{\;x}$$

ß0 = Y-intercept values or starting values of a slope.

Step 7. Multiple linear regression will be obtained from the slopes (ß1, ß2, ß3, ß4 and ß5) and Y-intercept values (ß0), from the (7), (8).

(7) $$Y={ß}_{1}x_{1}+{ß}_{2}x_{2}+\;{ß}_{3}x_{3}+{ß}_{4}x_{4}+{ß}_{5}x_{5}+{ß}_{0}$$

(8) $$Y={ß}_{1}\;d+{ß}_{2}\;s+\;{ß}_{3}\;ds+{ß}_{4}\;rc+{ß}_{5}\;r+{ß}_{0}$$

#### Testing model for AI-based DRM

Step 1: To remove noise from the feature matrix, normalization or the noise elimination technique is applied to the testing features.

Step 2: The predicted values are obtained by training the multiple linear regression. Model after the feature values have been normalized.

Step 3: Predicted model validation can be carried out through the mean squared error of the dynamic radio (M.S.E.D.R. ), mean absolute error of the dynamic radio (M.A.E.D.R. ), and root mean square error of dynamic radio (M.S.E.D.R. ) calculated from 9.

(9) $$\text{MAEDR}=\;\frac{1}{2m}\;\sum_{i=1}^{m}\left(t_{i}-\hat{\;t\;}\;\right){\;}^{2}$$

where $t_{i}$ represents the actual value and where t represents the predicted value.

Suppose that the M.A.E.D.R. value is minimal from the above formula calculation. This indicates that the accuracy of prediction for the required transmission power for any given distance is excellent. The total sum of error (T.S.E.) is calculated within the data error or variation of the data calculated from 10. It helps to calculate $R^{2}$ in calculated from 11.

(10) $$TSE=\frac{1}{2m}\;\sum_{i=1}^{m}\left(t_{i}-\;\bar{t}\;\right){\;}^{2}$$

(11) $$MSEDR\;or\;R^{2}=\frac{SSE}{TSE}$$

If the value of $R^{2}$ is one from the above formula, the prediction model is more effective. The coefficient of determination is another name for the root mean square error of the dynamic power.

### Method validation

#### Proposed protocol simulation and results analysis

**Fig. 6:**
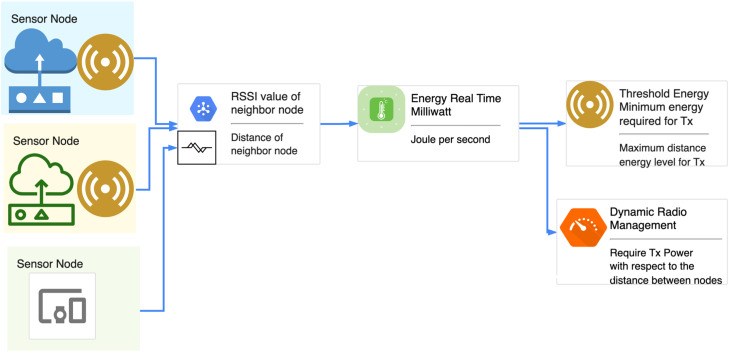
Architecture model for input of the AI-based DRM Protocol.

The proposed protocol of Section 6 is implemented via MATLAB MathWorks for 2000 rounds. A total of 110 sensors are deployed in the simulation as unstructured and heterogeneous WSNs, as shown in Fig. 6. If any sensor nodes want to communicate with the sink, the source node has to create a link via the propagation model explained in Section 3.2 and then send the packet via the proposed algorithm explained in Section 5. The parameters used in the proposed protocol simulation are described in Table 1. Algorithm 1

**Fig. 7:**
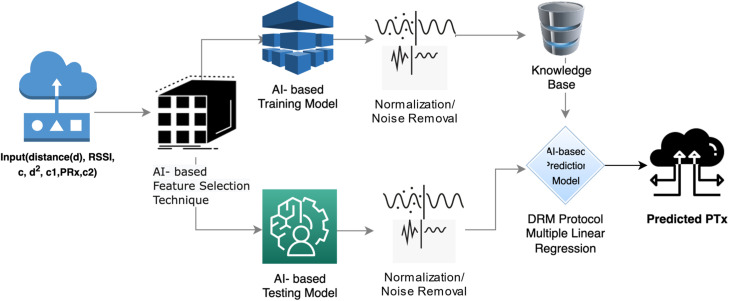
Overall architecture of the AI-based DRM prediction model.

**Table 1:** Simulation parameters.

DRM Parameters	DRM Measurements	DRM Parameters	DRM Measurements
Sensor nodes in DRM	110	Size of Packets	1024 bits
DRM simulation area	100 × 100 m2	Communication Range	30 m
Sink coordinators	50,120	Traffic Pattern	CBR

**Algorithm 1:** Dynamic Radio Power Consumption Algorithm.

**Input:** RSSI and distance d. **Output:** Dynamic Power Consumption for TX	
**Begin**	
1. Calculate the distance d between the sensor and its neighbour node.	
2. (If d ≤ Max_Tx_Range)	
	Calculate PTX according to the Free Space Path model.
3. If (PTX ≤ Max_PTX) && (PTX ≥ Min_PTX)	
	Check the threshold values and Apply PTX for transmission.
4. Sensor node energy consumption calculation	
4.1 total_energy_consumption=0; //Energy consumption when no event occurs 4.2 idle_energy_consumption = IdlePower * IdleTime; 4.3 total_energy_consumption += idle_energy_consumption; //Energy consumption while the upcoming event is sensed by the sensor 4.4 sensing_energy_consumption = SensingPower * SensingTime; 4.5 total_energy_consumption += sensing_energy_consumption; //Energy consumption while transmitting data to the next node 4.6 PTX_energy = PTX_power * PTX_time; 4.7 total_energy_consumption += PTX_energy; //Energy consumption while transmitting data to the next node 4.8 PRX_energy = PTX_power * PRX_time; 4.9 total_energy_consumption += PRX_energy; //Energy consumption per bit of packet size 4.10 total_energy_consumption += data_size * 0.01;	
else destination_node is out of range;	
5. WSN energy consumption calculation	
5.1 WSN_total_energy_consumption=0; 5.2 for i = 0 to n WSN_total_energy_consumption += total_energy_consumption [i];	
6. Total WSN energy consumption calculation	
6.1 WSN.rerouting_factor: 1.2, WSN.overhead_energy: 0.5; 6.2 WSN energy consumption during rerouting or link failure 6.3 WSN_total_energy_consumption *= WSN.rerouting_factors; 6.4 WSN energy consumption during overhead or heavy traffic 6.5 WSN_total_energy_consumption *= WSN.overhead_factors;	
7.End	

**Fig. 8:**
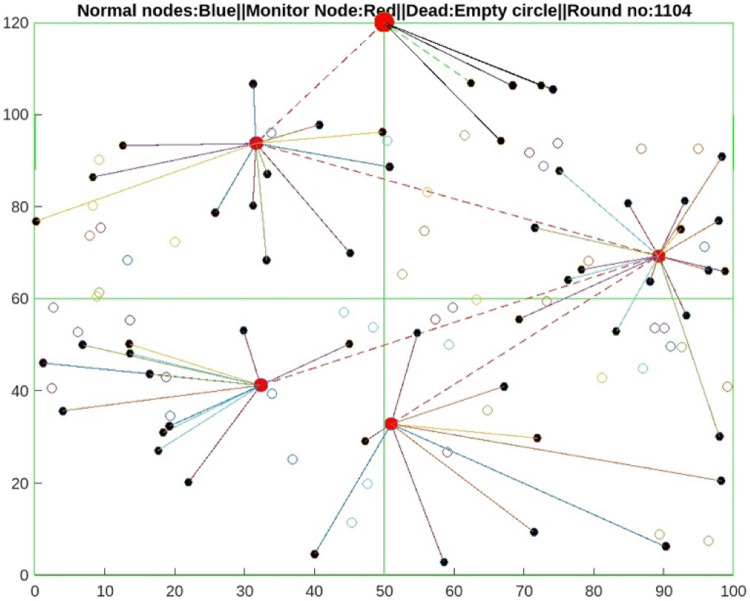
Alive nodes after 1104 rounds.

**Fig. 9:**
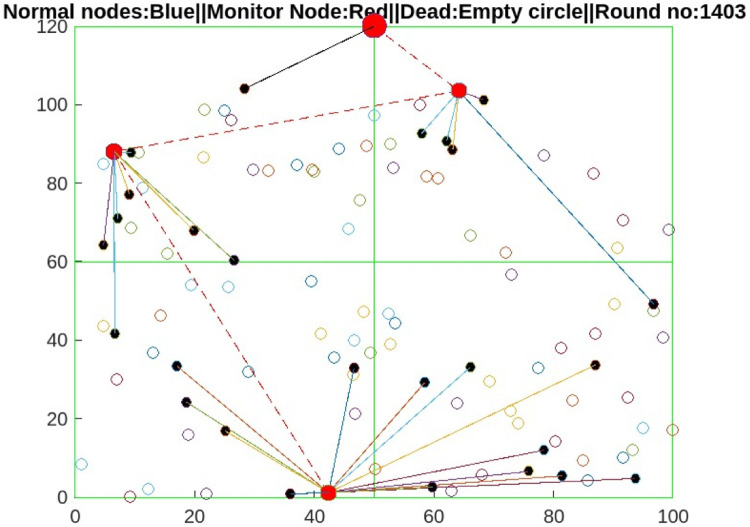
Alive nodes after 1403 rounds.

The proposed AI-DRM protocol is evaluated via a comparison of two recent energy-efficient protocols of 2024, the ARORA protocol and the EACHS-B2SPNN protocol [48]. Compared with the HBA , EFO [44], ROA [45], and ARO techniques [46], the ARORA Protocol [47] yields the best result and improves the lifetime of WSNs. All three ARORA protocols, the EACHS-B2SPNN protocol, and the proposed AI-DRM protocol are simulated via MATLAB. The simulation results are observed after 2000 rounds; the simulation shows that no nodes have sufficient residual energy to participate in transmission, so all nodes are declared dead nodes, as shown in Fig. 8, Fig. 9, and the results are stored in Table 2, Table 3, which contains the number of live sensors, the number of CH formations, and the residual energy of the WSN.

**Table 2:** Comparison of simulation results.

No. of Rounds	EACHs-B2SPNN			ARORA			Proposed AI-DRM		
	Alive Nodes	Residual Energy %	No. of CHs	Alive Nodes	Residual Energy%	No. of CHs	Alive Nodes	Residual Energy%	No. of CHs
100	110	90	7	110	90	13	110	96	4
200	109	76	6	110	78	12	110	90	4
300	108	66	11	110	64	15	110	80	4
400	107	54	5	110	56	8	110	70	4
500	106	42	12	110	44	14	110	62	4
600	105	32	4	106	34	6	110	52	4
700	95	22	11	104	28	11	110	44	4
800	80	16	9	100	18	9	110	38	4
900	67	2	12	78	6	12	90	30	4
1000	25	0	2	38	2	2	76	24	4
1100	10	0	0	15	0	0	60	20	4
1200	2	0	0	5	0	0	55	16	4
1500	0	0	0	0	0	0	38	4	1
2000	0	0	0	0	0	0	0	0	0

**Table 3:** AI-based DRM protocol comparison with recent works on Energy and Reliability.

Sl. No.	Protocol	FRE	EC	MER	MEC	PDR	NR
1	AI-based DRM	21600	5.88E+06	25797	4203	0	1400
2	IK-MACHES Protocol	145.43	3.27E+07	1966.99	23362.142	83.95	1400
3	PDBAC-LEACH Protocol	57.68	3.31E+07	5597.68	23635.239	89.9	1400
4	MAACO Routing Protocol	16588	9.39E+06	23289.65	6710.35	0	1400

Fig. 8 shows that 50% of the nodes are alive after 1100 rounds of communication in the proposed AI-DRM protocol, but no nodes are alive in the ARORA protocol or the EACHS-B2SPNN protocol after 1100 rounds. Fig. 9 shows that in the proposed protocol, 25% of the nodes have residual energy to communicate effectively until 1403 rounds without dropping packets. The proposed method can enhance the lifetime of WSNs by 28%.

**Fig. 10:**
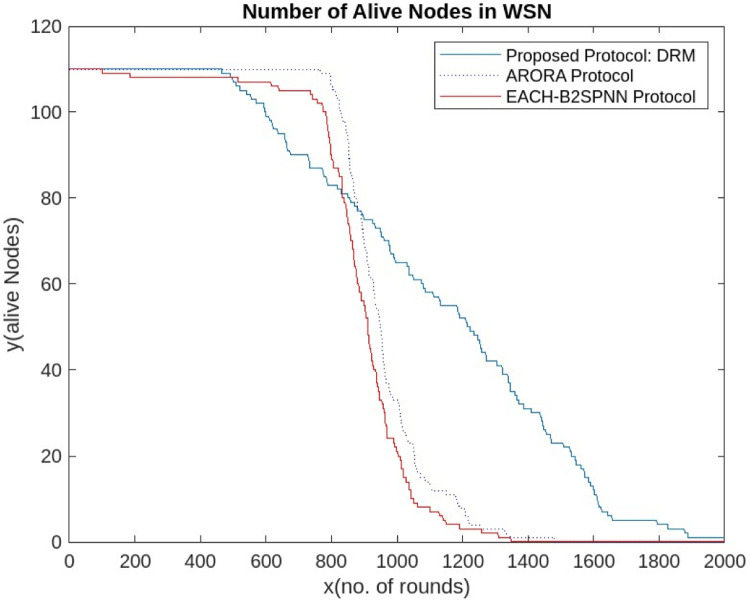
Comparison of the No. of Alive Sensors.

**Fig. 11:**
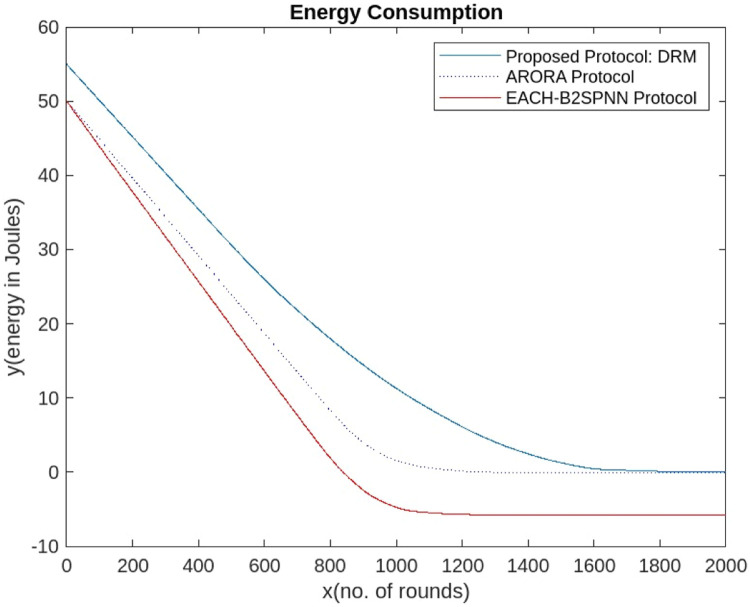
Comparison of residual energy.

The graph in Fig. 10 shows that after 1100 rounds of simulation, the sensors in the ARORA protocol [47] and the EACHS-B2SPNN protocol have very few live sensors [48], and no more transmission can be performed. However, the 1400 rounds effectively eliminate communication faults and packet drops. Similarly, Fig. 11 shows that the proposed AI-DRM protocol has better residual energy, which can lead to better throughput, as shown in Fig. 13, and a better lifetime for the WSN.

**Fig. 12:**
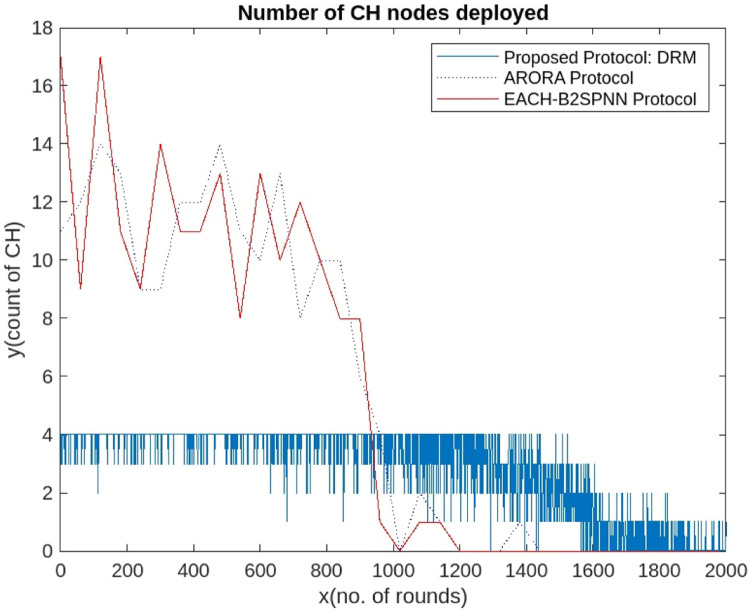
Comparison of No. CHs.

Compared with the ARORA protocol and the EACHS-B2SPNN protocol, the proposed AI-DRM protocol has a constant number of CH formations on the basis of the residual energy level of the nodes, which results in reliable and stable communication from source to sink via CHs, as shown in Fig. 12.

**Fig. 13:**
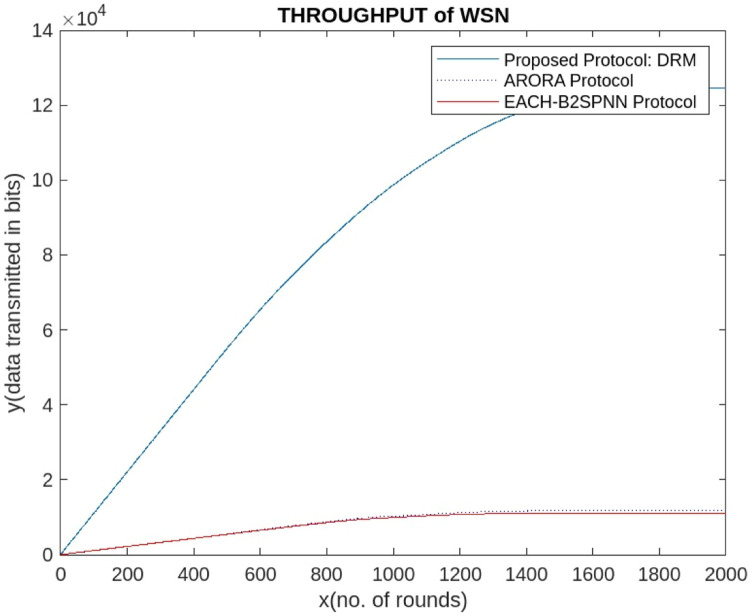
Comparison of throughputs.

Table 3 illustrates the results of AI-based DRM Protocol in comparison with the IK-MACHES Protocol [49], the PDBAC-LEACH Protocol [50], and the MAACO Routing Protocol [51]. The comparison is based on the following parameters: final residual energy, Energy Consumption, Mean residual energy, Mean energy consumption, and packet drop percentage.

**Fig. 14:**
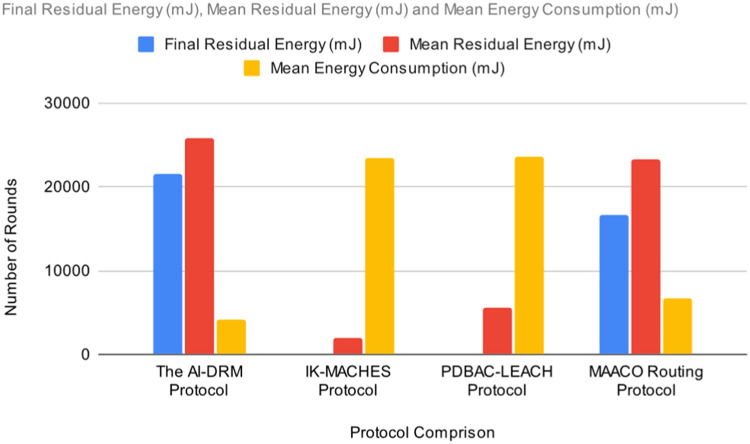
Result comparison of Final residual energy.

**Fig. 15:**
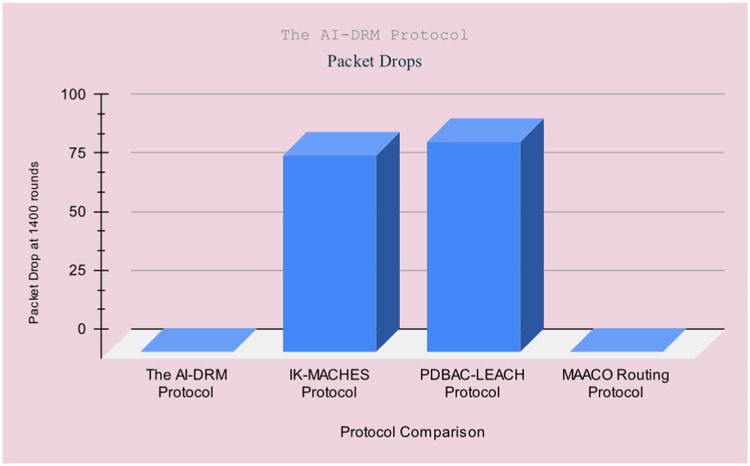
Result comparison of Packet Drop Percentage Mean residual energy and Mean energy consumption.

**Fig. 16:**
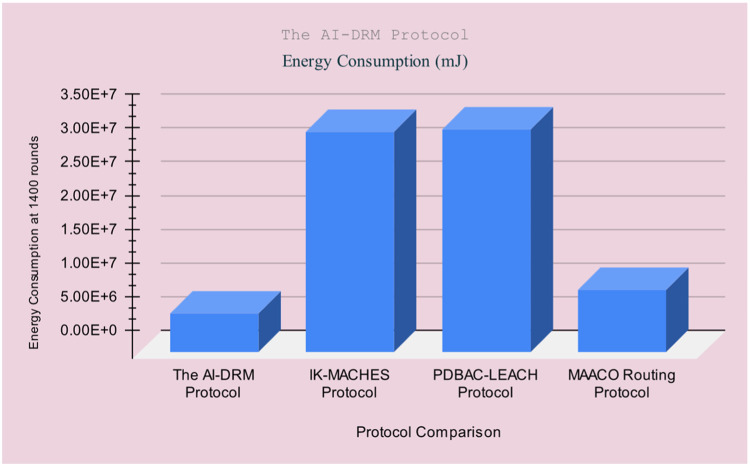
Result comparison of Energy Consumption.

The results indicate that the AI-based DRM Protocol as shown in Table 3. This significant improvement in performance marks a notable advancement. FRE: Final Residual Energy (mJ), EC: Energy Consumption (mJ), MER: Mean Residual Energy (mJ), MEC: Mean Energy Consumption (mJ), PDR: Packet Drop Ratio %, NR: Number of Rounds. Fig. 14 to 16 illustrates the Table 3 visualisation.

## Conclusion

The Dynamic Radio Management Protocol proves energy enhance of sensor nodes and the sensor network in the AI-based DRM Protocol It calculates the transmission power based on the distance of the source node to its neighbour nodes, enabling it to reach the destination and thereby reducing the power consumption of each sensor node, thereby doubling the lifetime of the best existing technique. Simultaneously, the protocol contributes to extending network longevity, increasing throughput and packet delivery ratio while reducing packet loss, latency, and jitter. Compared to existing work, which has less than 25% alive nodes, it also achieves more than 47% alive nodes, indicating that it can communicate with neighbour nodes even after 3000 rounds of communication. Comparatively, the AI-based DRM Protocol of WSNs utilises only 55% of the energy, whereas the current work's energy consumption accounts for over 90% of the total energy of WSNs in various simulation scenarios. The first dead node is discovered after 2662 communication rounds, whereas the existing study identifies it after 1837 communication rounds. The results demonstrate a significant enhancement in latency, featuring a minimum jitter of 5.2 ms, a minimum delay of 9 ms, an improved packet delivery rate of 98%, a reduced packet drop rate of 2%, a maximised throughput of 98%, and an average residual energy of 99% at 2000 rounds. The simulation findings reveal significant improvements in network reliability, energy efficiency, and resilience to node failures, as well as addressing security concerns within the network.

**Limitations**: In the future, work can be enhanced via dead node tracking and replacement of dead nodes with optimal routing protocols. Fig. 15

## Related research article

“None”

## Ethics statements

None

## CRediT author statement

Santosh Anand: Designed and implemented the protocol.

ANANTHA NARAYANAN V: Technical writing and testing of the work using hardware and simulation.

## Declaration of competing interest

The authors declare that they have no known competing financial interests or personal relationships that could have appeared to influence the work reported in this paper.

## Data Availability

No data was used for the research described in the article.
